# Pentraxin 3 depletion (PTX3 KD) inhibited myocardial fibrosis in heart failure after myocardial infarction

**DOI:** 10.18632/aging.204070

**Published:** 2022-05-06

**Authors:** Yufang Xu, Yiting Hu, Yanping Geng, Na Zhao, Caiyun Jia, Haojing Song, Wanjun Bai, Caihui Guo, Lili Wang, Yanhui Ni, Xiaoyong Qi

**Affiliations:** 1 Department of Internal Medicine, Hebei Medical University, Shijiazhuang, Hebei 050017, China; 2 Department of Pharmacy, Hebei General Hospital, Shijiazhuang 050051, Hebei, China; 3 Cardiovascular Medicine, Hebei General Hospital, Shijiazhuang 050051, Hebei, China

**Keywords:** heart failure, myocardial infarction, myocardial fibrosis, pentraxin 3, IL-6/STAT3 pathway

## Abstract

Background: HF is a common complication of MI. The underlying mechanisms of myocardial fibrosis in HF after MI are incompletely defined. Here, this study aims to investigate the role of PTX3 KD in HF after MI.

Methods: Bioinformatics analysis based on GSE86569 dataset was performed to explore the potential role of PTX3 in HF. Male C57/BL6J mice were administered with lentiviral vector encoding PTX3 KD or empty vector, and then underwent either coronary ligation or sham surgery. Echocardiography, Masson staining, and immunofluorescence counterstaining were conducted to evaluate the cardiac function and fibrosis. Cardiac fibroblasts were isolated and transfected with lentiviral vector encoding PTX3 KD *in vitro* to verify the *in vivo* findings.

Results: Bioinformatics analysis based on GSE86569 revealed the aberrant expression of PTX3 in HF patients. Echocardiography showed that PTX3 KD reversed the HF-induced cardiac dysfunction with better cardiac function parameters. Masson staining demonstrated that the obvious infarct and high fibrosis ratio in HF mice were remarkably improved after PTX3 KD. Immunofluorescence staining indicated that the HF-induced increase expression of α-SMA was significantly suppressed by PTX3 KD. Additionally, both *in vivo* and *in vitro* results confirmed that PTX3 KD decreased the fibrosis-related up-regulation of collagen I, collagen III, and p-STAT3. However, the result was opposite after IL-6 treatment.

Conclusions: PTX3 KD protects the cardiac function and counteracts the myocardial fibrosis by down-regulating IL-6/STAT3 pathway in HF.

## INTRODUCTION

Heart failure (HF) is a prevalent side effect of myocardial infarction (MI) over the world [1]. HF is a staggering clinical and public health concern, according to epidemiological research, with a prevalence of more than 23 million people globally [2]. HF is associated with significant morbidity and hospitalizations, even markedly increases the risk of death after MI by at least 3 to 4-fold [3]. Despite remarkable advancement in reducing HF-related mortality, hospitalizations for HF remain frequent and rates of readmissions continue to rise [2]. Efforts to promote myocardial repair have failed to translate into clinical therapies [4]. To prevent hospitalizations for HF after MI, a comprehensive research in its pathological mechanisms is imperative.

Recently, the importance of myocardial fibrosis in development and progression of HF has been highlighted [5]. The abnormal increase of cardiac fibroblasts and alterations of the cardiac extracellular matrix (ECM) result in cardiac fibrosis [6]. Myocardial fibrosis is mainly manifested as collagen synthesis, decreased collagen degradation and fibrotic scarring [7]. Eventually, myocardial fibrosis results in the alterations of myocardial architecture, facilitating the progression of cardiac dysfunction, inducing arrhythmias, and affecting the courses and outcomes of HF [6]. Moreover, myocardial fibrosis is an important determinant of pathologic hypertrophy in HF, also associated with disease severity and outcomes [8]. However, the underlying mechanisms of myocardial fibrosis in HF after MI are incompletely defined.

Pentraxin 3 (PTX3) is a stromal and myeloid cell-produced pentraxin that has been discovered as a cognate molecule of C reactive protein (CRP) [9]. It functions as a multifunctional protein that regulates inflammation, innate immunity, and ECM organization and remodelling [10]. PTX3, a novel inflammatory marker, has been shown to grow fast in HF, reflecting the degree of tissue damage and predicting the risk of mortality [11, 12]. However, little is known about PTX3’s molecular mechanism in HF, particularly in myocardial fibrosis. Focusing on novel pharmacological targets for cardiac fibrosis prevention can potentially improve patient care. Here, by bioinformatics analysis and post-MI HF mouse modelling, this study aims to verify the role of PTX3 in myocardial fibrosis and its potential downstream pathway.

## MATERIALS AND METHODS

### Bioinformatics analysis

GSE86569 dataset was downloaded from the Gene Expression Omnibus (GEO) database (http://www.ncbi.nlm.nih.gov/geo/). The raw microarray data was transformed into expressions using the EdgeR package. Kaplan-Meier survival curve analysis and the log-rank test were used to compare the overall survival (OS) between patients with HF and normal participants. RNA-seq data of shPTX3-transfected cardiomyocytes were retrieved from the GEO database to identify the differentially expressed genes (DEGs). Protein-protein interaction network was analysed by Cytoscape software. Gene Set Enrichment Analysis (GSEA) was performed in the Category package (version 2.10.1). Gene Ontology (GO) and Kyoto Encyclopedia of Genes and Genomes (KEGG) pathway analysis of DEGs was carried out using online tool DAVID. Enriched pathways were identified by Fisher’s exact test according to the cut-off criterion of adjusted *p*<0.05.

### Lentivirus construction

Mouse IL-6 over-expression (OE) lentivirus, >NM_031168.2|Il6[Mouse]|CDS 636bp, and the sequence: Atg aag ttc ctc tct gca aga gac ttc cat cca gtt gcc ttc ttg gga ctg atg ctg gtg aca acc acg gcc ttc cct act tca caa gtc cgg aga gga gac ttc aca gag gat acc actc cca aca gac ctg tct ata cca ctt cac aag tcg gag gct taa tta cac atg ttc tct ggg aaa tcg tgg aa atg ag aaa aga gtt gtg caa tgg caa ttc tga ttg tat gaa caa cga tga tgc act tgc aga aaa caa tct gaa actt cca gag ata caa aga aat gat gga tgc tac caa ac tgg ata taa tca gga aat ttg cct att gaa aat ttc ctc tgg tct tct gga gta cca tag cta cct gga gta cat gaa gaa caa ctt aaa aga taa caa gaa aga caa agc cag agt cct tca gag aga tac aga aac tct aat tca tat ctt caa cca aga ggt aaa aga ttt ac ata aaa tag tcc ttc cta ccc caa ttt cca atg ctc tcc taa cag ata agc tgg agt cac aga agg agt ggc taa gga cca aga cca tcc aat tca tct tga aat cac ttg aag aa ttt cta aaa gtc act ttg aga tct act cgg caa acc tag. PTX3 knock down lentivirus, transcript: NM_ 008987.3 GENE ID:19288, 3 shRNA knockdown sequences were designed as follows: si1 GGG ACA AGC TGT TCA TCA TGC; si2 GGA GGA GCC CAG TAT GTT TCT; si3 GGA GAA GGT AGT TGT GTA ATT. Viral vector: GL107 pSLenti-EF1-EGFP-P2A-Puro-CMV-MCS-3xFLAG-WPRE, GL107 was set as control. And the lentivirus was constructed by Syngentech Co., LTD (Beijing, China).

### Animal modelling and treatment

A total of 30 specific pathogen-free C57/BL6J mice (male, 7-8 weeks old, 20-22 g) were purchased from Beijing Vital River Laboratory Animal Technology Co., Ltd. (Beijing, China). All mice were raised in the standard laboratory environment (25±2° C, 60±5% humidity and 12/12 h light/dark cycle) and had free access to food and water. All procedures involving animals were performed in accordance with NIH Guide for the Care and Use of Laboratory Animals and authorized by the Institutional Review Board of Hebei General Hospital. Mice were randomly allocated at 1:1:1 into three groups, namely Sham group, control group and PTX3 KD group. Ten mice in sham group received a single bolus of empty vector, (1 × 10^7^ PFU/mouse) and then underwent the sham surgery. For the remaining 20 mice, a single bolus of empty vector (model group) or lentiviral vector encoding PTX3 KD (PTX3 KD group, 1 × 10^7^ PFU/mouse) were injected into the heart, 7days before surgery. While the other mice experiments for Sham group, Model group, and IL-6 OE group were injected empty control vector (1contr^7^ PFU/mouse) and IL-6-OE (1 grou^7^ PFU/mouse) lentivirus 7 days before surgery. Next, these 20 mice underwent the ligation of left coronary artery, according to the methods described previously [13]. After 8-week feeding, all mice were used for subsequent analysis.

### Echocardiography and electrocardiogram evaluation

After 8-week feeding, the echocardiography was conducted in all groups using Vevo2100 VisualSonics (VisualSonics Inc., Canada) under light anaesthesia with vaporize isoflurane (4.5%) and spontaneous respiration. The 2D targeted M-mode tracings were recorded at a paper speed of 50 mm/s. Under anaesthesia with 1.5% isoflurane, left ventricular (LV) pressure was measured by a micro manometer-tipped catheter in the LV. According to the echocardiography, LV anterior wall thickness at end-diastolic (LVAWd) or end-systolic (LVAWs), LV internal diameter at end-diastolic (LVIDd) or end-systolic (LVIDs) at three cardiac cycles were calculated. Ejection fraction (EF) and fraction shortening (FS) were recorded as well. The apical four chamber view was taken, the mitral valve flow spectrum was measured by PWD, and the peak velocity of early diastolic flow (E) and late diastolic flow (A) were recorded; M-mode color Doppler images of mitral valve orifice were recorded to obtain the left ventricular blood flow propagation velocity in the early diastole; After obtaining satisfactory parasternal long axis, color blood flow was used to locate the left atrial entrance of pulmonary vein, obtain the spectrum of pulmonary vein, and record the peak velocity of pulmonary vein in systole (PVS), positive peak velocity of pulmonary vein in diastole (PVD) and negative peak velocity of pulmonary vein in atrial systole (PVA) to evaluate the diastolic functions. ECG were used to diagnose the myocardial infarction, mice were put in a supine position and connected to three electrodes: one on the left foreleg, one on the right foreleg, and one on the right hind leg and then the ECG was recorded [14].

### Tissue preparation and Masson staining

After *in vivo* echocardiographic measurements, all mice were anesthetized with 3% chloral hydrate and the heart was excised. Then, the heart was dissected into the right and left ventricles. The middle ring of LV was harvested, fixed in 4% paraformaldehyde, embedded in paraffin and sliced into 5 μm-thick sections. Then, the sections were stained with Masson’s trichrome according to standard protocol. The fibrosis ratio was expressed as the percentage of interstitial fibrosis in the total LV area.

### Detection of MI area by 2,3,5-triphenyltetrazolium chloride (TTC) staining

The myocardial tissues were quickly frozen in a refrigerator at -20° C for 10 min, sliced into 5 sections of about 2 mm in thickness, and incubated in 2% TTC PBS by tin foil paper away from light at 37° C for 30 min, followed by staining on the other side. Normal tissues were stained red, while infarcted tissues were not stained. Then the sections were fixed with 4% paraformaldehyde for 12 h and photographed, and the photos were analysed using ImageJ software. Finally, the MI area was calculated.

### Wheat germ agglutinin (WGA) staining

The WGA antibody (1:150) was diluted and added to the section, after incubation at 37° C in dark for 1 h, wash off the antibody and then DAPI agent were used for nuclear staining. The images were collected by fluorescence electron microscope (Nikon, Japan). After the film, the transverse muscle fibers were counted by IPP 6.0 image analysis software. Cross-sectional area, 150 ~ 200 cardiac muscle fibers per tissue [15].

### Immunofluorescence (IF) staining

IF counterstaining was performed according to standard procedures. Briefly, paraffin tissue sections were deparaffinized in xylene, and rehydrated in graded ethanol before antigen retrieval in citrate buffer (10 mM, at 98° C) for 20 min. After cooling down, the sections were washed in PBS with 0.3% Triton X-100 and blocked with 5% donkey normal serum for 1 h at room temperature. Then, the sections were incubated overnight with rabbit anti-cardiac troponin T (ab209813, Abcam) and mouse anti-α skeletal muscle actin (α-SMA) primary antibodies (ab28052, Abcam). After washing with PBS, the sections were stained with goat anti-rabbit IgG H&L (Alexa Fluor® 488) (ab150077, Abcam) and goat anti-mouse IgG H&L secondary antibodies (Alexa Fluor® 488) (ab150113, Abcam); for PTX3-Immunofluorescence staining, the PTX3 ((ab125007, 1:50 dilution) and goat anti-rabbit IgG H&L (Alexa Fluor® 488) were used. Then the sections were counterstained with 4’,6-diamidino-2-phenylindole (DAPI, blue) to stain nuclei. Images were captured under a confocal microscope.

### Western blotting assay

One portion of heart tissues was homogenized to extract the protein. Whole-cell lysates were extracted by RIPA lysis buffer (ThermoFisher Scientific, USA). After the quantification of total proteins, lysates were separated by SDS-PAGE and transferred to the PVDF membrane. The PVDF membrane was then sealed with 5% skim milk at 37° C for 1 h, followed by incubation with specific primary antibodies to PTX3 (ab125007, 1:1000), IL-6 (ab6672, 1:500), p-STAT3 (ab76315, 1:50), Type I collagen (ab34710, 1:1000), Type III collagen (ab6310, 1:500) or GAPDH (ab181602, 1:2000) at 4° C for 12 h. Subsequently, the PVDF membrane was incubated with HRP-conjugated anti-rabbit IgG secondary antibody at 37° C for 1 h. Protein bands were developed by enhanced chemiluminescence. Finally, the band intensity was analysed by IPP 6.0 software.

### Preparation of cardiac fibroblasts

Cardiac fibroblasts were isolated from C57/BL6J neonatal mice by digestion with collagenase II [14]. Briefly, heart tissues were dissected into pieces (1 mm^3^) and digested 5 times (15 min/time, 37° C) with 0.1% (w/v) collagenase type II in Hank’s Balanced Salt Solution (HBSS). The resulting cell suspension was centrifuged at 1000 r/min for 5 min and washed in PBS. Then the cells were incubated with red blood cell lysis buffer (eBioscience, San Diego, CA, USA) to remove red blood cells. After centrifugation, the cells were re-suspended with DMEM containing 20% fetal bovine serum (FBS). The culture flask was placed in a 5% CO_2_ incubator at 37° C for 90 min, then the supernatant was absorbed to remove non-adherent fibroblasts and dead cells, and 5 mL of complete medium was added for further culture. Cardiac fibroblasts were cultured from the adherent cells.

### Vimentin identification and α-SMA of myocardial fiber

A total of 10^5^ second-generation primary cardiac fibroblasts were inoculated into 6-well plates. The expression of Vimentin and α-SMA in cardiac fibroblasts was detected by IF. The positive cells were counted and photographed under a fluorescence microscope, and the positive cells/plate >80% were used for our experiments.

### Treatment of cardiac fibroblasts

After 3-generation subculture, cardiac fibroblasts were subjected to different treatment. Fibroblasts in control group were maintained in DMEM. For PTX3 KD, fibroblasts were transfected with lentiviral vector encoding PTX3 KD (PTX3-KD group), using empty vector as a negative control (PTX3-NC group). Then transfected fibroblasts were cultured in DMEM containing 0.1 μM Ang II and 10 ng/mL TGF-β at 37° C. Meanwhile, to verify the downstream pathway, the transfected fibroblasts (PTX3-KD+IL-6 group) or its negative control (PTX3-NC+IL-6 group) was exposed to 20 ng/mL IL-6 (an activator for STAT3 signalling) in DMEM containing 10 ng/mL TGF-β. After culture for 72 h, the fibroblasts were used for cell viability and Western blotting assay.

### Cell viability measurement

Cell viability was evaluated by Cell Counting Kit-8 (CCK-8) assay according to manufacturer’s instructions (Beyotime, Beijing, China). The treated fibroblasts in the logarithmic phase were seeded in a 96-well plate at a density of 3×10^4^ cells/well. After incubation for 24, 48 and 72 h, 10% (w/v) CCK-8 solution was added into each well and incubated for another 2 h. Optical density (OD) was detected at 450 nm using a microplate reader.

### Statistical analysis

Statistical analysis was performed by GraphPad Prism 6.0 (GraphPad Software, San Diego, CA, USA). Data were presented as mean ± standard error of mean (SEM) in each experiment. Multiple comparisons were made by one-way ANOVA with post hoc Bonferroni correction. *p*<0.05 was considered significant.

## RESULTS

### Bioinformatics analysis indicated the potential association between PTX3 and HF progression

**Figure 1:**
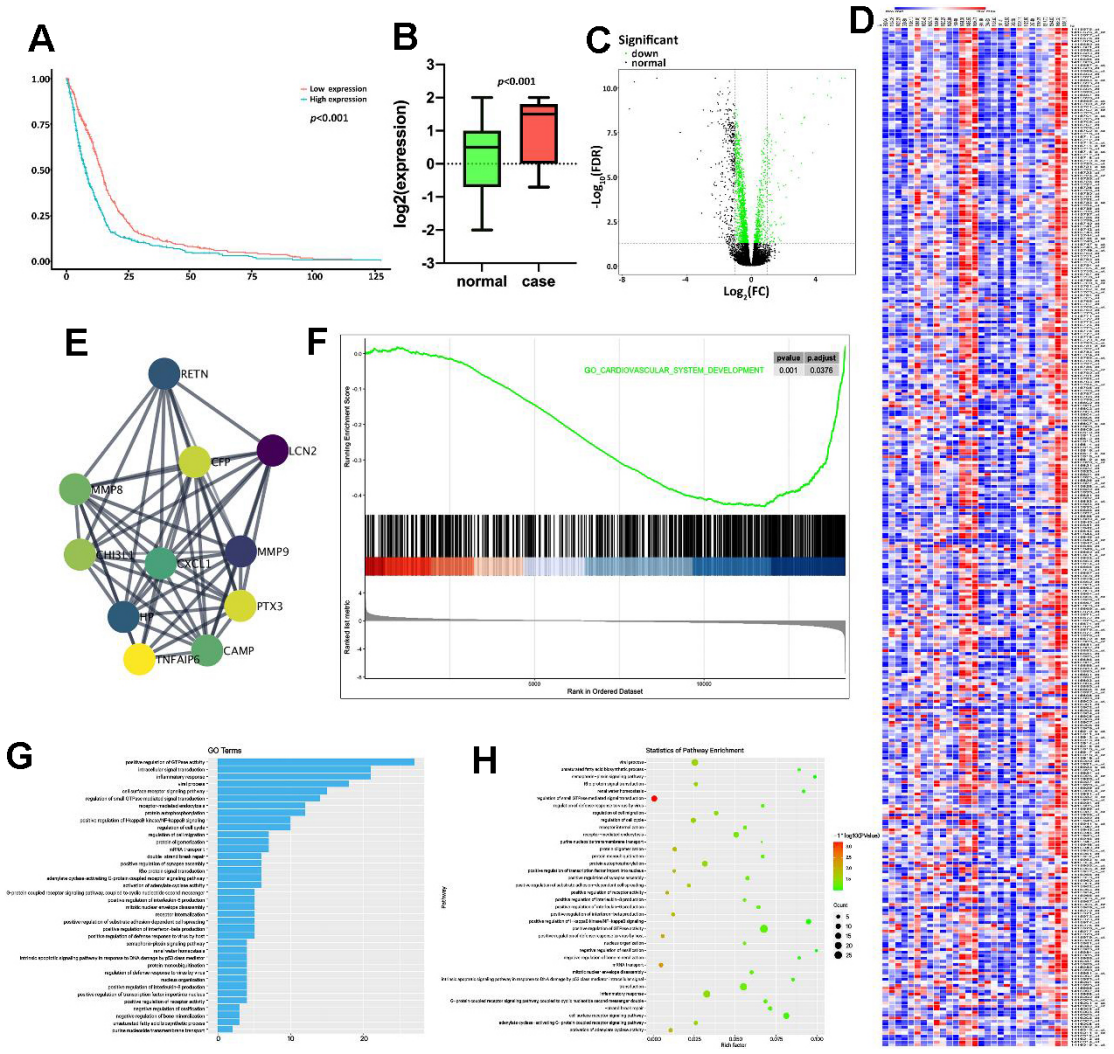
**Bioinformatics analysis indicated the potential association between PTX3 and HF progression.** (**A**) Kaplan-Meier analysis in GEO database showed that the up-regulation of PTX3 was associated with a lower overall survival rate. (**B**) PTX3 in tissues from HF patients was up-regulated compared with that in normal tissues. (**C**) Volcano plot and (**D**) heatmap of the PTX3-induced DEGs; red indicates the up-regulated genes and blue indicates the down-regulated genes. (**E**) Protein-protein interaction network of DEGs. (**F**) GSEA showed a significant correlation between PTX3 expression and the pathways related to IL-6 production. (**G**) GO analysis and (**H**) KEGG analysis revealed the representative enrichment pathways.

Firstly, the GSE86569 dataset consisting of HF samples was obtained to assess the role of PTX3 in HF. Survival analysis showed that the high expression level of PTX3 was linked to a lower overall survival rate (Figure 1A). Moreover, we found that the PTX3 was upregulated in patients with HF compared to healthy controls (Figure 1B), suggesting that PTX3 may play a role in HF progression. The DEGs were shown in the volcano plot and heatmap (Figure 1C, 1D). A total of 325 DEGs were identified, of which 155 were up-regulated and 170 were down-regulated. Through PPI network analysis, we found that PTX3 interacted with cAMP and matrix metalloproteinases (MMPs), including MMP8 and MMP9 (Figure 1E). Moreover, GSEA showed that PTX3 expression was substantially linked to the pathways related to IL6 production (Figure 1F). GO and KEGG analysis demonstrated that the IL-6 production pathway, inflammatory response pathway, GTPase activity pathway, and cell surface receptor pathway were all enriched in HF (Figure 1G, 1H). In short, the bioinformatics analysis indicated that PTX3 may be involved in HF progression, and correlated with the inflammatory response in HF.

### PTX3 KD improved the cardiac functions in murine HF after MI

**Figure 2:**
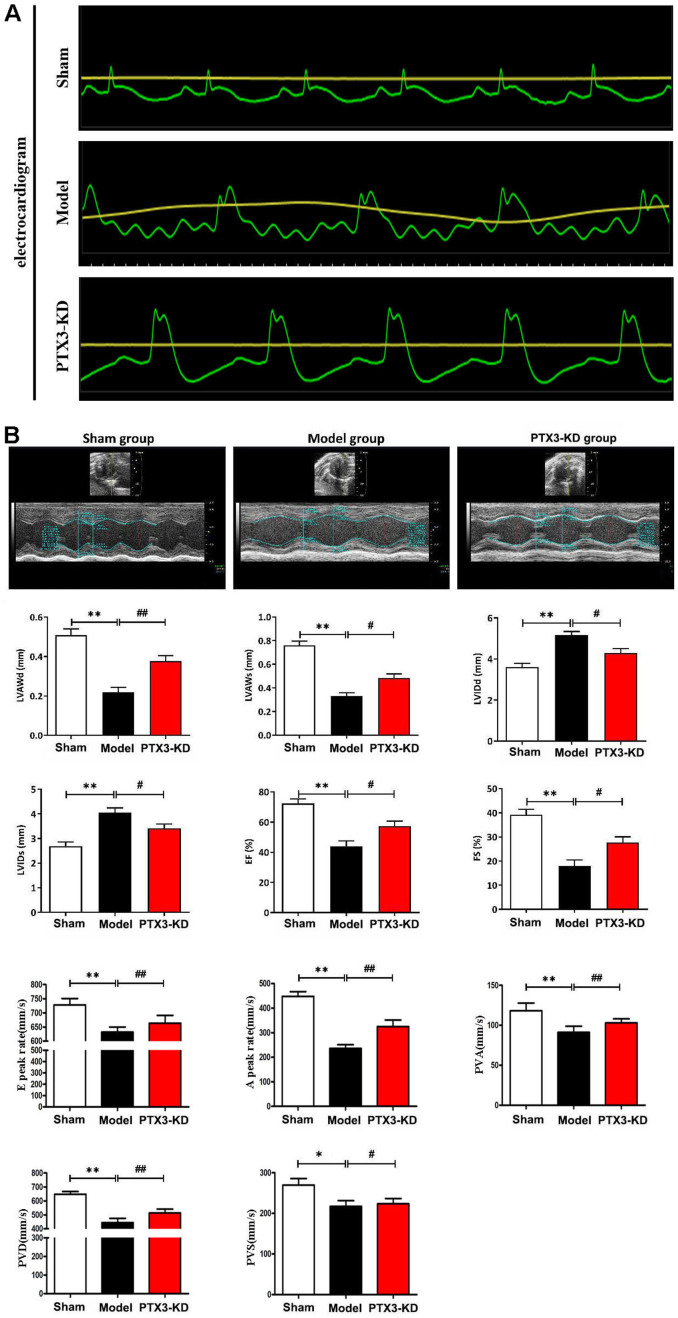
**PTX3 KD improved the cardiac functions in murine HF after MI.** (**A**) Electrocardiogram results confirmed that the animal model was successfully constructed. (**B**) Representative M-mode echocardiogram of LV in the parasternal short-axis view obtained from mice in each group. Echocardiographic parameters (LVAWd, LVAWs, LVIDd, LVIDs, ES and FS as well as E peak rate, A peak rate, PVA, PVD, PVS) at three cardiac cycles were recorded. LVAWd: left ventricular anterior wall thickness at end-diastolic; LVAWs: left ventricular anterior wall thickness at end-systolic; LVIDd: left ventricular internal diameter at end-diastolic; LVIDs: left ventricular internal diameter at end-systolic; EF: ejection fraction; FS: fraction shortening; PVD: Positive peak velocity of pulmonary vein in diastole; PVS: Peak systolic velocity of pulmonary vein; PVA: Negative peak velocity of pulmonary vein during atrial contraction. Control group vs. PTX3-NC group, *p<0.05, ** *p*<0.01; PTX3-NC group vs. PTX3-KD group, ^#^ *p*<0.05, ^##^ *p*<0.01.

To verify the role of PTX3 KD in the improvement of HF after MI, coronary artery ligation was performed in mice. The mice who survived 6 h after coronary artery ligation were given either a lentiviral vector encoding PTX3 KD (PTX3-KD group, n=10) or an empty vector (model group, n=10) for induction. All of the animals made it to the end of the research period. Control (n=10), model (n=10), and PTX3-KD (n=10) animals were all subjected to M-mode echocardiography. Electrocardiogram results showed the MI model is shown in Figure 2A. Representative echocardiography images of different mice are shown in Figure 2B. Echocardiographic measurements demonstrated that mice in PTX3-KD group had lower LVAWd, LVAWs, ES and FS, but higher LVIDd and LVIDs compared with those in model group, as well as PTX3-KD improved the E and A peak rate, PVA, PVD, PVS compared with Model-group (Figure 2B), indicating the PTX3-KD Successfully improved the systolic and diastolic function of the heart of murine HF after MI. It was found that the changes of echocardiographic parameters in murine HF after MI were all reversed after PTX3 KD, demonstrating that PTX3 KD improves the cardiac functions.

### PTX3 KD reduced the infarct areas and cardiomyocyte hypertrophy in MI mice

**Figure 3:**
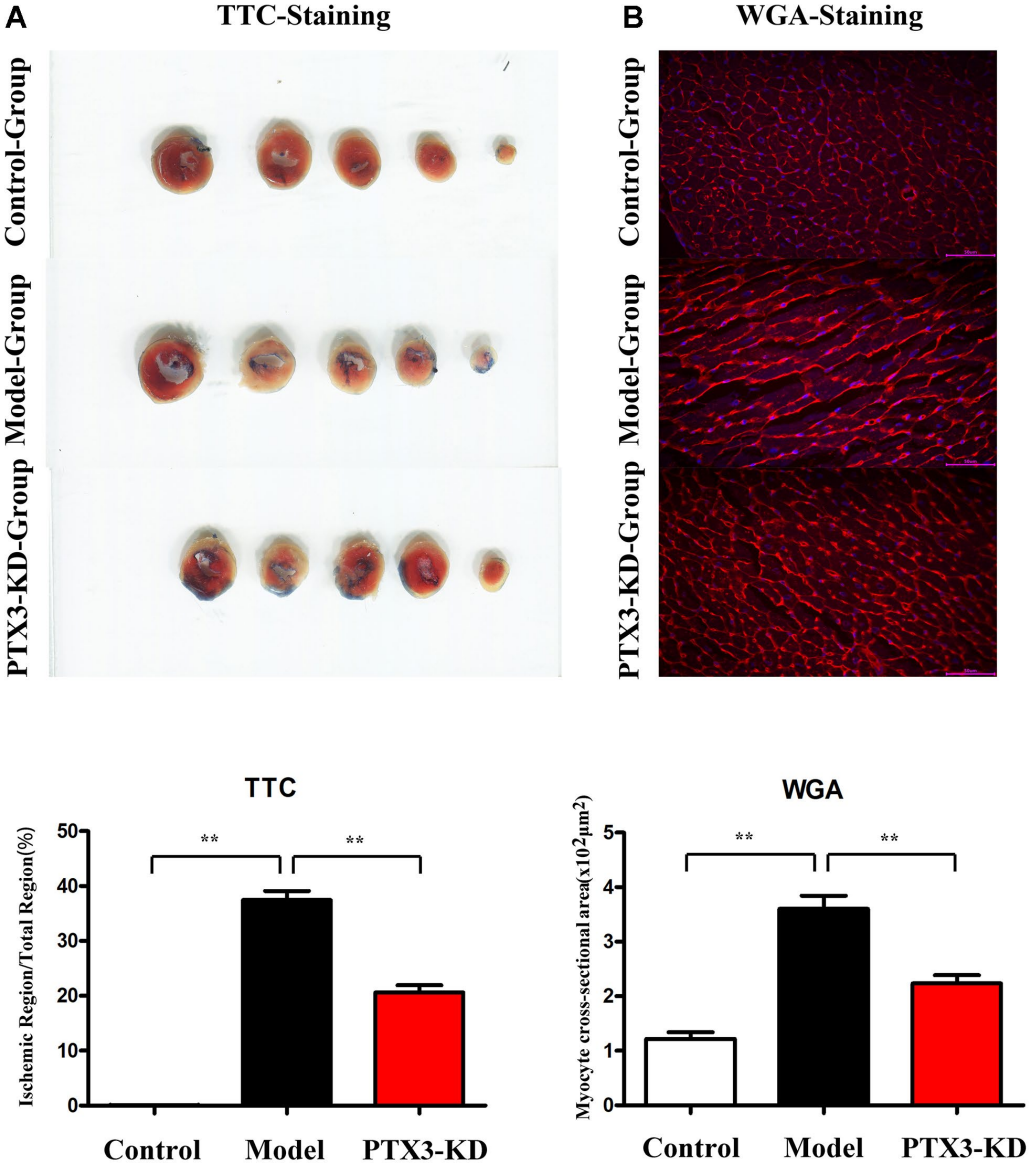
**PTX3 KD decreased infarcted areas as well as myocardial hypertrophy in MI of mice.** (**A**) TTC staining images in each group, PTX3 KD decreased infarcted areas of heart after MI. (**B**) Representative WGA staining in each group, PTX3 KD inhibited the degree of myocardial hypertrophy in mice of MI. *p<0.05, ** *p*<0.01, *** *p*<0.001.

In HF mice, an evident infarct that extended into the anterolateral wall was identified; however, after PTX3 KD, the infarct area was significantly reduced (Figure 3A). TTC results showed that compared with the HF group, the area of myocardial infarction in PIX3 KD group mice was significantly reduced. WGA staining was used to assess the areas of cross-sectional area (CSA) of cardiomyocytes, the PTX3 KD suppressed the areas of CSA, indicating its inhibitory roles for cardiomyocyte hypertrophy.

### PTX3 KD counteracted myocardial fibrosis by down-regulating IL-6/STAT3 pathway in murine HF after MI

**Figure 4:**
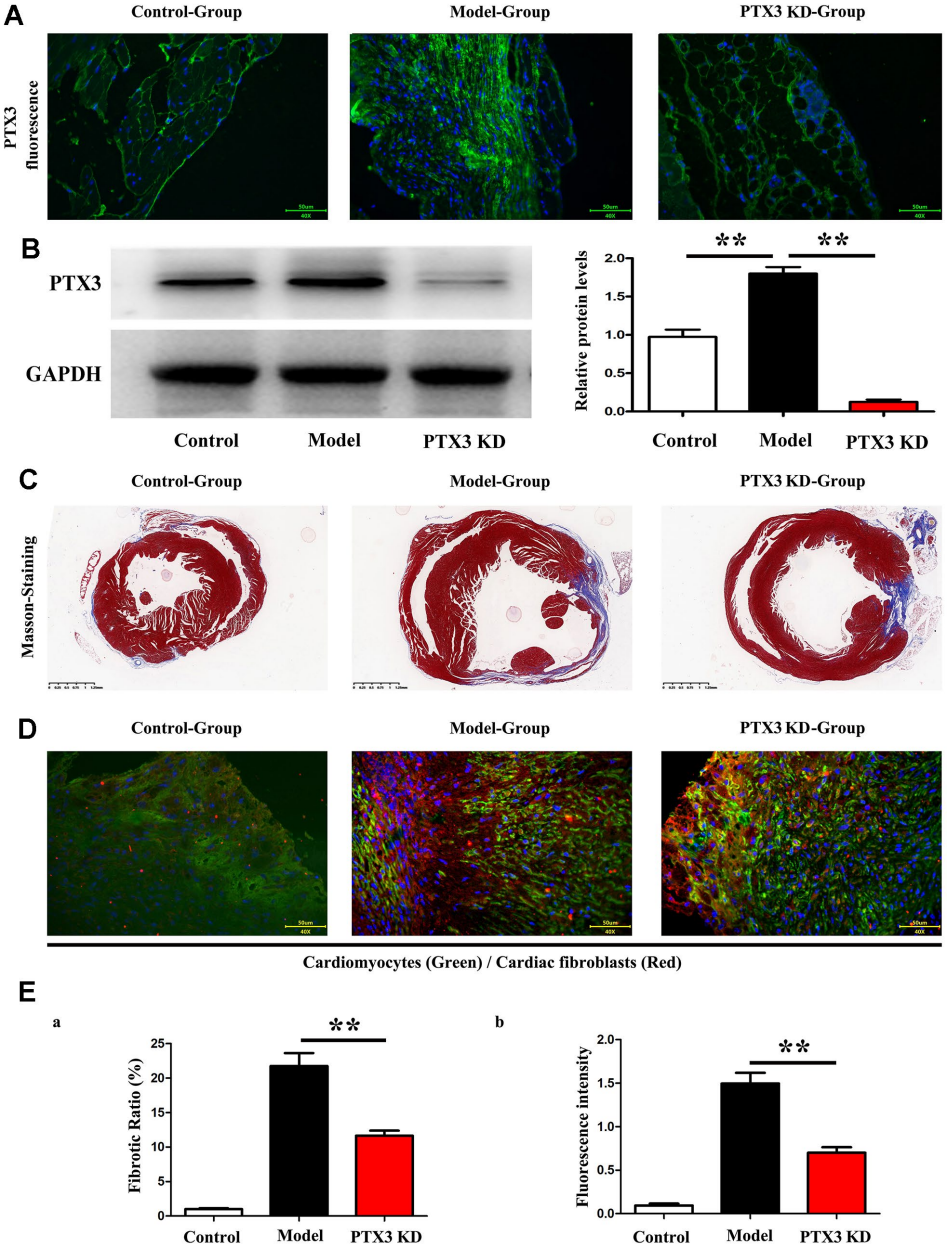
**PTX3 KD counteracted myocardial fibrosis by down-regulating IL-6/STAT3 pathway in murine HF after MI.** (**A**) Representative Masson staining images in each group. (**B**) The fibrosis ratio was quantified by the percentage of interstitial fibrosis in the total LV area in Masson staining and expressed as the average of all sections. (**C**) Representative IF counterstaining images in each group (×40). Cardiomyocytes were stained with cardiac troponin T (green) and cardiac fibroblasts were stained with α-SMA (red). (**D**) Fluorescence intensity of α-SMA was quantified. (**E**) Western blotting was performed to measure the protein expression. Control group vs. PTX3-NC group, *p<0.05, ** *p*<0.01, *** *p*<0.001; PTX3-NC group vs. PTX3-KD group, ^#^ *p*<0.05, ^##^ *p*<0.01, ^###^ *p*<0.001.

**Figure 5:**
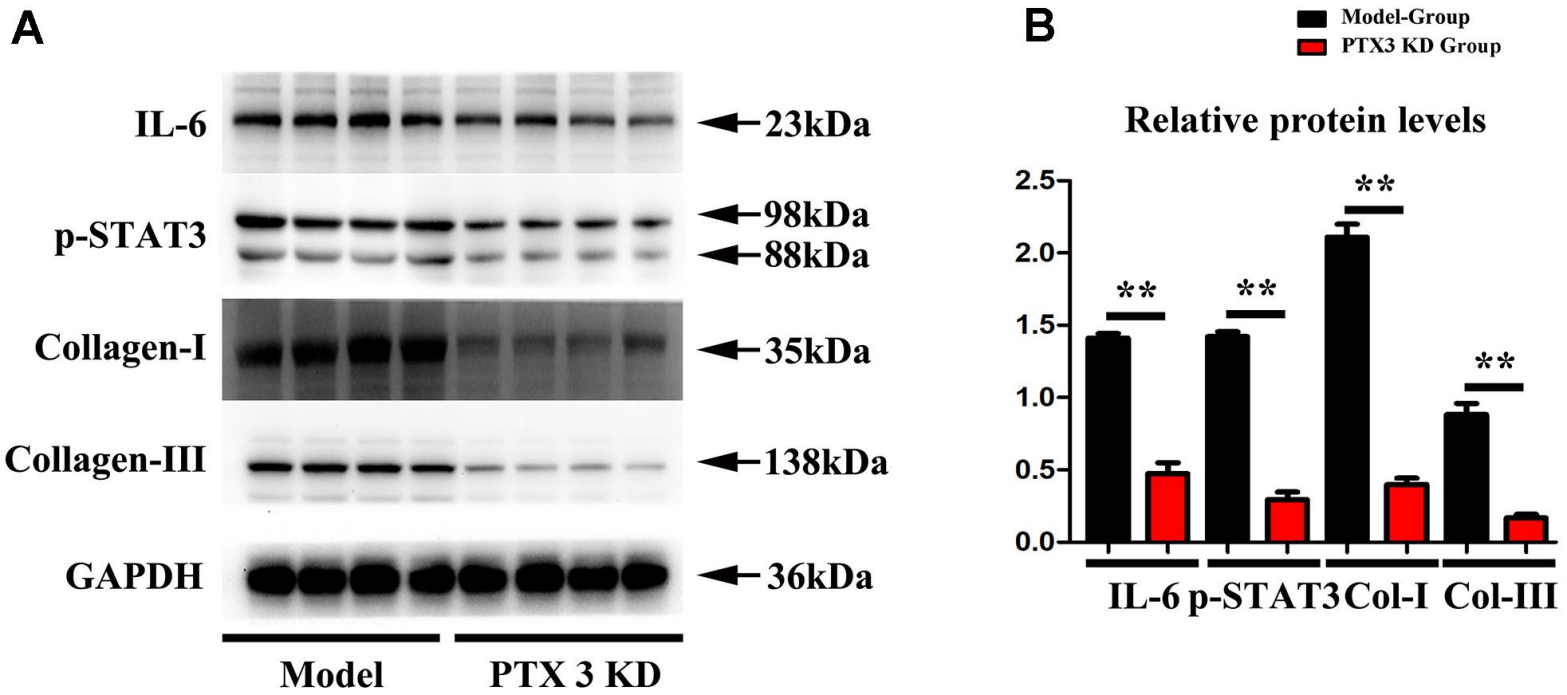
**PTX3 KD successfully suppressed the expression of IL-6/STAT3 signals in MI.** (**A**, **B**) Western blotting was performed to measure the IL-6 and p-STAT as well as fibrosis-associated proteins collagen-I/III expression. Control group vs. PTX3-NC group, *p<0.05, ** *p*<0.01, *** *p*<0.001; PTX3-NC group vs. PTX3-KD group, ^#^ *p*<0.05, ^##^ *p*<0.01, ^###^ *p*<0.001.

Subsequently, Immunofluorescence staining results showed the successful inhibition of PTX3 protein expression after PTX3-KD lentivirus treatment, as shown in Figure 4A, and moreover, the western blot results showed the significant decreased expression of PTX3 in PTX3 KD group when compared with control and model group, shown in Figure 4B. Masson staining were conducted to assess the effects of PTX3 KD on myocardial fibrosis in murine HF after MI. Figure 3C showed the transverse LV sections (mid-cavity) stained with Masson’s trichrome. Consistent with Masson staining showed that the myocardial fibrosis was significantly reduced after PTX3 KD. Through the pathological images, the fibrosis ratio was estimated to provide a basis of comparison. As shown in Figure 4D, the fibrosis ratio in PTX3-KD group was significantly lower than that in model group. Besides, Figure 4D shows the IF staining of LV sections. Consistent with the results of Masson staining, the fluorescence intensity of cardiac fibroblasts (α-SMA, red fluorescence) after PTX3 KD was obviously decreased compared with that in model group (Figure 4D). Type I collagen (collagen I) and type III collagen (collagen III), as the major components of ECM, are related to the fibrosis. Along with the PTX3 KD, both collagen I and collagen III were down-regulated (Figure 4E), suggesting the alteration of ECM. Based on the potential association between PTX3 and IL6 revealed by bioinformatics analysis, we next investigated the expression of IL-6 and p-STAT3 in tissues. As expected, Western blotting revealed that both IL-6 and p-STAT3 were down-regulated in PTX3-KD group compared with those in model group (Figure 5A, 5B), indicating that PTX3 can inhibit the IL-6/STAT3 pathway. Overall, these above results demonstrate that PTX3 KD counteracts myocardial fibrosis by down-regulating IL-6/STAT3 pathway in murine HF after MI.

### IL-6 STAT3 signal accelerated the progression of fibrosis in MI

**Figure 6:**
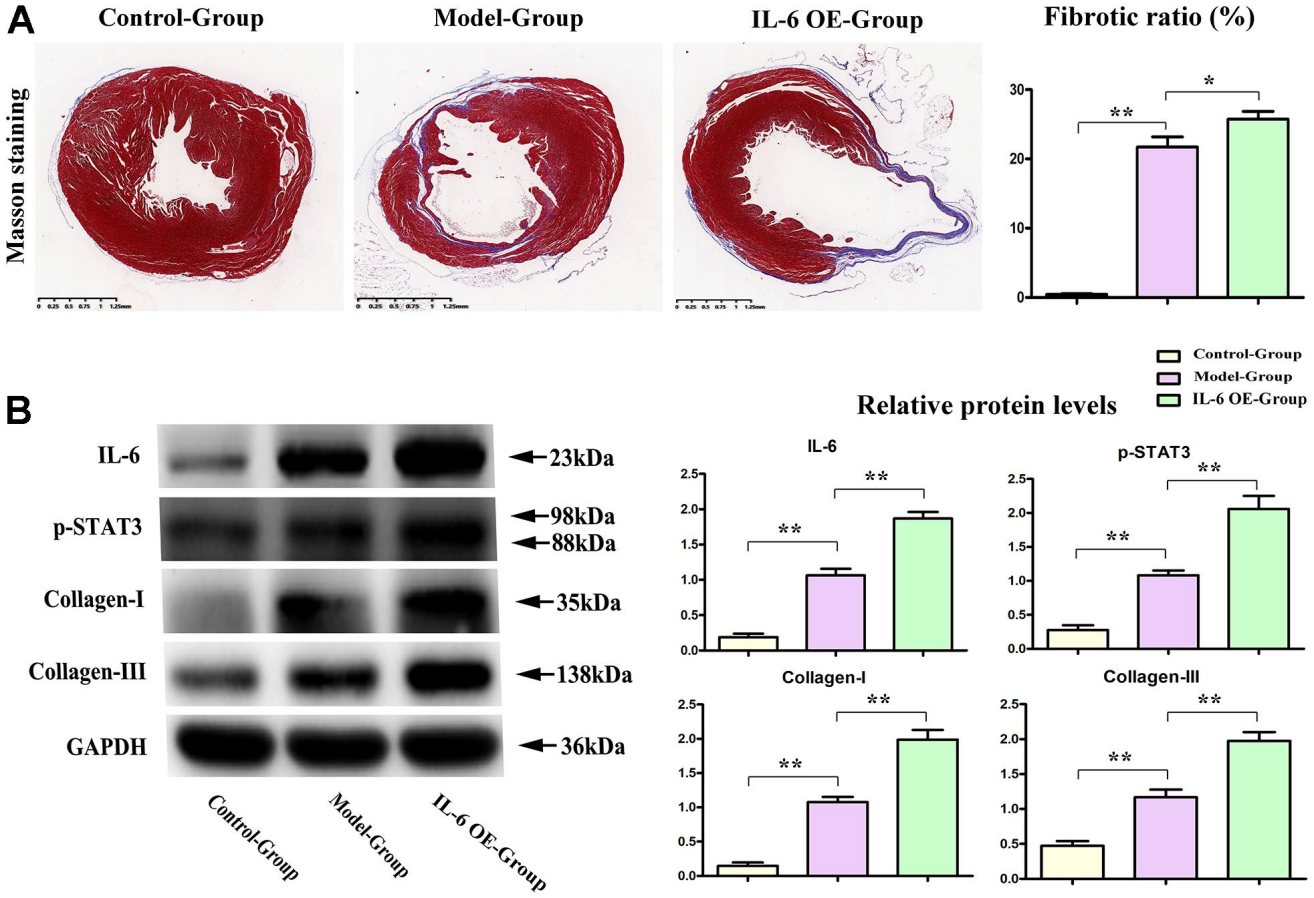
**IL-6 STAT3 signal pathway positively accelerated the fibrotic progression of MI.** (**A**) IL-6 OE significantly increased the fibrotic areas in MI vs model group. (**B**) Western blot results showed the obvious increased expression of IL-6, p-STAT3, and collagen I/III in IL-6 OE group mice vs Model group mice. *p<0.05, ** *p*<0.01, *** *p*<0.001.

To determine the effects of IL-6/STAT3 signals in fibrosis of MI, the IL-6 OE lentivirus were used to specifically active STAT3 (Phosphorylated form) in heart, and the results showed that over expression of IL-6 could increase the fibrotic areas after MI and shown in Figure 6A. The relative proteins of IL-6 and phosphorylated STAT3 (p-STAT3) as well as fibrosis associated proteins: collagen I/III were tested by western blot and our results showed the over expression of IL-6 could significantly aggravate and promote the expression of IL-6, p-STAT3 as well as collagen-I/III, shown in Figure 6B, these results intensely indicated the IL-6/ STAT3 signal plays the pivotal roles in fibrosis of MI in hearts, and PTX3’ roles on IL-6/STAT3 signals could effectively influence the progression of fibrosis in MI.

### PTX3 KD inhibited the viability of cardiac fibroblasts by down-regulating IL-6/STAT3 pathway *in vitro*

**Figure 7:**
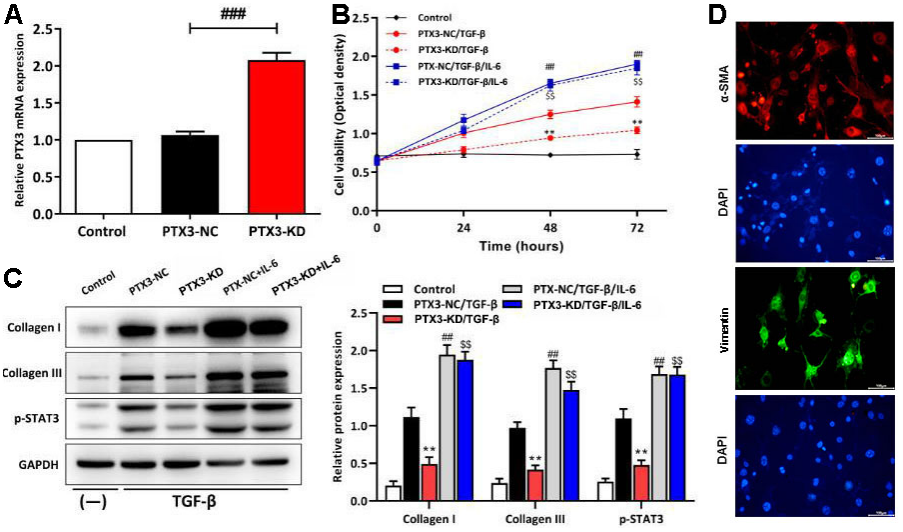
**PTX3 KD inhibited the viability of cardiac fibroblasts by down-regulating IL-6/STAT3 pathway *in vitro*.** (**A**) qRT-PCR results confirmed the successful PTX3 KD in cardiac fibroblasts. (**B**) The transfected fibroblasts were exposed to IL-6 in the presence of TGF-β and cultured for 72 h. CCK-8 assay was performed to measure the cell viability. (**C**) Western blotting was performed to measure the expression of collagen I, collagen III and p-STAT3. (**D**) Primary cardiac fibroblast climbing sheets were stained with immunofluorescence staining for a-SMA, specific marker. PTX3-NC/TGF-β group vs. PTX3-KD/TGF-β group, *p<0.05, ** *p*<0.01; PTX3-NC/TGF-β vs. PTX3-NC/TGF-β/IL-6 group, ^#^ *p*<0.05, ^##^ *p*<0.01; PTX3-KD/TGF-β vs. PTX3-KD/TGF-β/IL-6 group, ^$^ *p*<0.05, ^$$^ *p*<0.01.

To confirm the results of the *in vivo* studies, cardiac fibroblasts from C57/BL6J mice were extracted and transfected with PTX3 KD vector. As shown in Figure 7A, qRT-PCR showed the expression of PTX3 was the lowest in cardiac fibroblasts in PTX3-KD group. Next, under the stimulation of TGF-β (to stimulate fibrosis *in vitro*), transfected fibroblasts were treated with IL-6 (an activator for STAT3 signalling) to verify the role of IL-6/STAT3 pathway in the PTX3 KD-mediated cardioprotective effect. CCK-8 assay showed that the cell viability of cardiac fibroblasts with PTX3 KD was obviously lower than that of NC (Figure 7B). Conversely, the cell viability was significantly enhanced after IL-6 exposure (Figure 7B). Meanwhile, Western blotting revealed that the PTX3 KD down-regulated the expression of collagen I and collagen III, further supporting that PTX3 KD counteracts fibrosis (Figure 7C). In addition, after activating the IL-6/STAT3 pathway (shown as up-regulation of p-STAT3 in Figure 4C) by IL-6, we observed that the PTX3 KD-induced down-regulation of collagen I and collagen III was remarkably reversed (Figure 7C). Overall, these above results indicate that PTX3 KD inhibits the viability of cardiac fibroblasts by down-regulating the IL-6/STAT3 pathway. The fibroblast cells were identified by staining with a-SMA (red) and vimentin (green), the specific marker for fibroblast, these results were shown in Figure 7D.

## DISCUSSION

The present study demonstrated that PTX3 KD decreased the cardiac fibroblasts and altered the ECM by suppressing the expression collagen I and collagen III. It can be seen that PTX3 KD protects the cardiac function and counteracts the myocardial fibrosis in HF after MI. Additionally, we preliminarily proposed the potential mechanism that PTX3 KD exerted the cardioprotective effect through suppressing the IL-6/STAT3 pathway.

Plasma PTX3 is higher in individuals with MI according to previous clinical observations [15]. Mounting data suggests that PTX3 may serve as a prognostic marker in HF patients [12]. Consistent with these previous studies, the high expression of PTX3 in patients with HF was confirmed through bioinformatics analysis. Furthermore, PTX3 has been linked to the advancement of cardiovascular diseases (CVD) in a number of investigations [16]. As a result, we created HF animal models and measured cardiac functions to see how PTX3 KD affected them. The echocardiography results revealed that PTX3 KD improved the HF-caused cardiac dysfunction with better cardiac function parameters (higher LVAWd, LVAWs, ES and FS, but lower LVIDd and LVIDs), suggesting the recovery of LV function. PTX3 appears to play a role in inflammation and exacerbates tissue damage according to an increasing body of evidence [17]. In present study, given these above results, we preliminary confirmed that PTX3 KD played a cardioprotective role in HF mouse model.

Myocardial fibrosis is a significant component of cardiac remodelling that results in HF [18]. In present study, it was found that PTX3 KD resulted in a significant reduction in infarct size, accompanied by the decreased accumulation of α-SMA. α-SMA is a classical marker for cardiac fibroblasts [19], whose down-regulation indicates the decreased cardiac fibroblasts. Cardiac fibrosis is caused by an aberrant increase in cardiac fibroblasts and enhanced cardiac fibroblast activity, as previously observed [6]. Thus, our results suggested that the PTX3 KD decreased the number of cardiac fibroblasts *in vivo* and also inhibited the activity of cardiac fibroblasts *in vitro*, suggesting that PTX3 KD attenuates the myocardial fibrosis by inhibiting cardiac fibroblasts. Furthermore, PTX3 has been found in fibrotic regions co-localizing with collagen [20]. The excess of cardiac collagen type I synthesis and deposition has been found in the enhancement of myocardial fibrosis during the development of HF [21]. Meanwhile, type III collagen is required for collagen I fibrillogenesis, and the two collagens interact with each other during fibrillogenesis [22, 23]. Type I and III collagens are major components of the ECM, thus the pathological remodelling and excessive deposition of ECM leads to fibrotic scars [24, 25]. Thus, it is widely recognized that myocardial fibrosis also results from excessive ECM deposition [20]. Here, Western blotting demonstrated that PTX3 KD inhibited the expression of collagen I and III both *in vivo* and *in vitro*, further indicating that PTX3 KD regulates the ECM remodelling and decreases the fibrotic scars. Similar to our findings, lots of evidence has proposed that PTX3 is involved in regulation of tissue remodelling and repair [26]. Taking the above evidence together, it is concluded that PTX3 KD counteracts myocardial fibrosis in HF mouse models.

**Figure 8:**
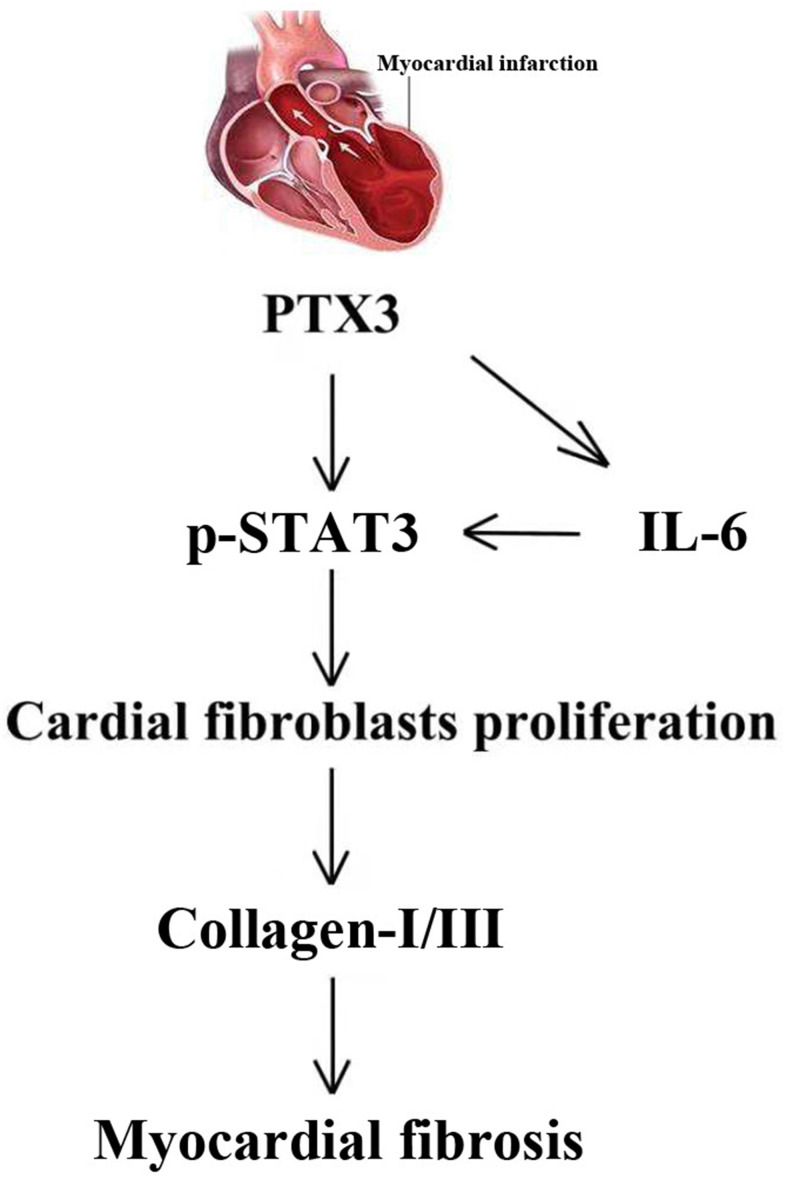
The mechanisms of PTX3 in myocardial fibrosis were shown schematically.

Through the GSEA analysis, it was found that PTX3 was significantly linked to the production of IL-6. Furthermore, IL-6 is a key activator of STAT3, a protein that is important in cardiac physiology and pathology [14]. As a result, it is hypothesized that PTX3 may play a role in the IL-6/STAT3 pathway. Recent evidence has suggested that IL-6 as one fibrogenic cytokine plays important roles in the fibrogenesis [27], and it binds to its receptor, leading to the recruitment and phosphorylation of STAT3 [28]. STAT3 promotes fibroblast activation and tissue fibrosis by integrating numerous pro-fibrotic signals [29]. In present study, on the one hand, Western blotting confirmed the downregulation of IL-6 and p-STAT3 caused by PTX3 KD, implicating that PTX3 can inhibit the IL-6/STAT3 pathway. On other hand, it was found that IL-6 exposure reversed the PTX3 KD-mediated increase in activity of cardiac fibroblasts and down-regulation of collagen I, collagen III, and p-STAT3, which further supported our hypothesis. Similarly, a recent has also suggested that PTX3 KD inhibits fibrosis through suppressing the IL-6/STAT3 pathway [13]. Thus, these results demonstrate that PTX3 KD counteracts myocardial fibrosis by inactivating the IL-6/STAT3 pathway, and the mechanisms of PTX3 were shown schematically in Figure 8.

## CONCLUSIONS

In summary, the study results demonstrate that PTX3 KD plays a cardioprotective role in HF mouse models. PTX3 can counteract the myocardial fibrosis by inhibiting the IL-6/STAT3 pathway, implicating that PTX3 KD may acts as a therapeutic target for HF after MI. Illustrating the molecular mechanism of PTX3 in myocardial fibrosis provides insights on the transfer of the new mechanistic knowledge on HF pathology into potential biomedical applications.
